# Editorial to the Special Issue “MicroRNA in Solid Tumor and Hematological Diseases”

**DOI:** 10.3390/biomedicines9111678

**Published:** 2021-11-12

**Authors:** Francesca Lovat

**Affiliations:** Department of Cancer Biology and Genetics and Comprehensive Cancer Center, The Ohio State University, Columbus, OH 43210, USA; francesca.lovat@osumc.edu

In the last two decades, the roles of microRNAs in the biology and progression of human cancer have been extensively studied; at present, these small non-coding RNAs are considered powerful gene regulators. microRNAs are involved in almost all biological and cellular processes, such as proliferation, differentiation and apoptosis [1]. Aberrant microRNA expression in cancer cells and in their microenvironment, and in body fluids such as serum or urine, has been linked to transformation, stemness, metastasis, resistance to chemotherapy and immune modulation in different tumor types, both solid tumor and hematological diseases.

This Special Issue includes eleven papers—five original manuscripts, six reviews and one systematic review—investigating the biological role of microRNAs in different tumor types and potential diagnostic and clinical approaches.

Lin and colleagues [2] presented a detailed overview of the role and regulatory mechanisms of microRNAs that control their dysregulated expression, focusing on solid tumors, including colorectal cancer, lung cancer, breast cancer, and liver cancer. Artemaki and co-workers [3] dissected the role of microRNAs in normal B-cell development and their deregulation in B-cell non-Hodgkin lymphomas (NHLs). Korac and colleagues [4] reported interesting details of miR-7’s roles in cancer biology and development. Mainly described as a tumor-suppressor, miR-7 can act as an oncomiR, underlining how microRNA expression is tissue- and microenvironment-specific. Okada and colleagues [5] reported the tumor-suppressive role of the miR-139 duplex (miR-139-5p and miR-139-3p) in renal cell carcinoma (RCC). The miR-139 duplex can modulate and potentially silence different oncogenes involved in the pathogenesis of RCC. Lee and colleagues [6] described the role of microRNAs in the regulation of Hippo-YAP/TAZ signaling in liver cancer. MicroRNAs, functioning as oncogenes and/or tumor-suppressors, can directly or indirectly modulate the Hippo-YAP/TAZ signaling pathway, leading to the development and progression of hepatic cancer.

The discovery of the potential role of microRNAs as a biomarker in the detection of the early stage of the disease represents one of the most promising developments in microRNA research. It is well documented that microRNAs are released into body fluids, and their detection represents a powerful noninvasive and sensitive method for early diagnosis. Aita and colleagues [7] analyzed serum from patients with early pancreatic ductal adenocarcinoma (PDAC) to identify a microRNA signature, to ensure early diagnosis and potentially predict prognosis. Kovynev and colleagues [8] identified a set of microRNAs that were able to detect the minimal residual disease (MRD) of acute leukemia (AL), regardless of myeloid (AML) or lymphoblastic leukemia (ALL) origin, compared with the hematopoietic conditions induced by non-tumor pathologies (NTPs) used as controls. In addition to early disease detection, microRNAs serve as useful candidate biomarkers to stratify patients with primary resistance to a specific targeted therapy and those who have developed acquired resistance. Angerilli and colleagues [9] reported an accurate description of microRNAs as mediators of resistance to a specific targeted therapy used in the treatment of gastrointestinal tumors, focusing on anti-EGFR, anti-HER2 and anti-VEGF antibodies, small-molecule tyrosine kinase inhibitors and immune checkpoint inhibitors. Dias and co-workers [10] described a microRNA signature capable of modulating the L-type amino acid transporter 1 (LAT1) and alanine–serine–cysteine transporter 2 (ASCT2) expression in colorectal cancer (CRC). Cancer cells require an increased intake of amino acid to maintain their proliferation rate; therefore, dysregulation of these two amino acid transporters plays an important role in CRC development. This microRNA set could represent an inhibitory tool for a potential therapeutic approach. Ravegnini and colleagues [11] presented a systematic review of circulating microRNAs that were correlated with therapy in epithelial ovarian cancers (EOCs). The final analysis pinpointed the miR-200 family as the potential biomarker in EOC. The miR-200 family has been described as being involved in the epithelial–mesenchymal transition (EMT) pathway, which promotes EOC progression and metastasis.

The results from studies of circulating microRNAs are often not reproducible due to a lack of accurate quantification of these molecules. The lack of standardization in the protocols has been identified as a possible cause of this issue, particularly the use of different strategies to normalize microRNA expression. Oto and colleagues [12] reported that miR-29c-3p represents the most stably expressed microRNA, and, therefore, the best normalizer in urine of bladder cancer (BC) patients. The discovery of this noninvasive stable reference will support future microRNA analysis among urine samples of BC patients.

All the research presented in this Special Issue provides an overview of the potential use of microRNAs to understand tumor biology and explain their roles as biomarkers to modulate the biological pathways that are critical for cancer development and progression, as well as providing useful insights to predict clinical outcomes or responses to therapy. We are only at the beginning of the journey to understanding and clinically applying the knowledge of microRNAs. We hope that the studies presented in this Special Issue can spark additional interest and trigger new, insightful research in this field in rapid expansion.

## Data Availability

Not applicable.

## References

[1] Lovat F., Valeri N., Croce C.M. (2011). MicroRNAs in the pathogenesis of cancer. Semin. Oncol..

[2] Lin Y.-C., Chen T.-H., Huang Y.-M., Wei P.-L., Lin J.-C. (2021). Involvement of microRNA in Solid Cancer: Role and Regulatory Mechanisms. Biomedicines.

[3] Artemaki P.I., Letsos P.A., Zoupa I.C., Katsaraki K., Karousi P., Papageorgiou S.G., Pappa V., Scorilas A., Kontos C.K. (2021). The Multifaceted Role and Utility of MicroRNAs in Indolent B-Cell Non-Hodgkin Lymphomas. Biomedicines.

[4] Korać P., Antica M., Matulić M. (2021). MiR-7 in Cancer Development. Biomedicines.

[5] Okada R., Goto Y., Yamada Y., Kato M., Asai S., Moriya S., Ichikawa T., Seki N. (2020). Regulation of Oncogenic Targets by the Tumor-Suppressive miR-139 Duplex (miR-139-5p and miR-139-3p) in Renal Cell Carcinoma. Biomedicines.

[6] Lee N.-H., Kim S.J., Hyun J. (2021). MicroRNAs Regulating Hippo-YAP Signaling in Liver Cancer. Biomedicines.

[7] Aita A., Millino C., Sperti C., Pacchioni B., Plebani M., De Pittà C., Basso D. (2021). Serum miRNA Profiling for Early PDAC Diagnosis and Prognosis: A Retrospective Study. Biomedicines.

[8] Kovynev I.B., Titov S.E., Ruzankin P.S., Agakishiev M.M., Veryaskina Y.A., Nedel’ko V.M., Pospelova T.I., Zhimulev I.F. (2020). Profiling 25 Bone Marrow microRNAs in Acute Leukemias and Secondary Nonleukemic Hematopoietic Conditions. Biomedicines.

[9] Angerilli V., Galuppini F., Businello G., Dal Santo L., Savarino E., Realdon S., Guzzardo V., Nicolè L., Lazzarin V., Lonardi S. (2021). MicroRNAs as Predictive Biomarkers of Resistance to Targeted Therapies in Gastrointestinal Tumors. Biomedicines.

[10] Dias F., Almeida C., Teixeira A.L., Morais M., Medeiros R. (2021). LAT1 and ASCT2 Related microRNAs as Potential New Therapeutic Agents against Colorectal Cancer Progression. Biomedicines.

[11] Ravegnini G., De Iaco P., Gorini F., Dondi G., Klooster I., De Crescenzo E., Bovicelli A., Hrelia P., Perrone A.M., Angelini S. (2021). Role of Circulating miRNAs in Therapeutic Response in Epithelial Ovarian Cancer: A Systematic Revision. Biomedicines.

[12] Oto J., Plana E., Fernández-Pardo Á., Cana F., Martínez-Sarmiento M., Vera-Donoso C.D., España F., Medina P. (2020). Identification of miR-29c-3p as a Robust Normalizer for Urine microRNA Studies in Bladder Cancer. Biomedicines.

